# MiR129-5p-loaded exosomes suppress seizure-associated neurodegeneration in status epilepticus model mice by inhibiting HMGB1/TLR4-mediated neuroinflammation

**DOI:** 10.1007/s11033-024-09215-z

**Published:** 2024-02-08

**Authors:** Tengfei Liu, Haiyan Liu, Siyi Xue, Lijie Xiao, Jing Xu, Shuyan Tong, Xiu’e Wei

**Affiliations:** 1 Department of Neurology, The Second Affiliated Hospital of Xuzhou Medical University, Xuzhou, 221006 Jiangsu China; 2 School of Medical Imaging, Xuzhou Medical University, Xuzhou, 221004 Jiangsu China

**Keywords:** Exosome, miR129-5p, Kainic acid, Status epilepticus, Neuroinflammation

## Abstract

**Background:**

Neuroinflammation contributes to both epileptogenesis and the associated neurodegeneration, so regulation of inflammatory signaling is a potential strategy for suppressing epilepsy development and pathological progression. Exosomes are enriched in microRNAs (miRNAs), considered as vital communication tools between cells, which have been proven as potential therapeutic method for neurological disease. Here, we investigated the role of miR129-5p-loaded mesenchymal stem cell (MSC)-derived exosomes in status epilepticus (SE) mice model.

**Methods:**

Mice were divided into four groups: untreated control (CON group), kainic acid (KA)-induced SE groups (KA group), control exosome injection (KA + Exo-con group), miR129-5p-loaded exosome injection (KA + Exo-miR129-5p group). Hippocampal expression levels of miR129-5p, HMGB1, and TLR4 were compared among groups. Nissl and Fluoro-jade B staining were conducted to evaluate neuronal damage. In addition, immunofluorescence staining for IBA-1 and GFAP was performed to assess glial cell activation, and inflammatory factor content was determined by ELISA. Hippocampal neurogenesis was assessed by BrdU staining.

**Results:**

The expression of HMGB1 was increased after KA-induced SE and peaking at 48 h, while hippocampal miR129-5p expression decreased in SE mice. Exo-miR129-5p injection reversed KA-induced upregulation of hippocampal HMGB1 and TLR4, alleviated neuronal damage in the hippocampal CA3, reduced IBA-1 + and GFAP + staining intensity, suppressed SE-associated increases in inflammatory factors, and decreased BrdU + cell number in dentate gyrus.

**Conclusions:**

Exosomes loaded with miR129-5p can protect neurons against SE-mediated degeneration by inhibiting the pro-inflammatory HMGB1/TLR4 signaling axis.

**Graphical Abstract:**

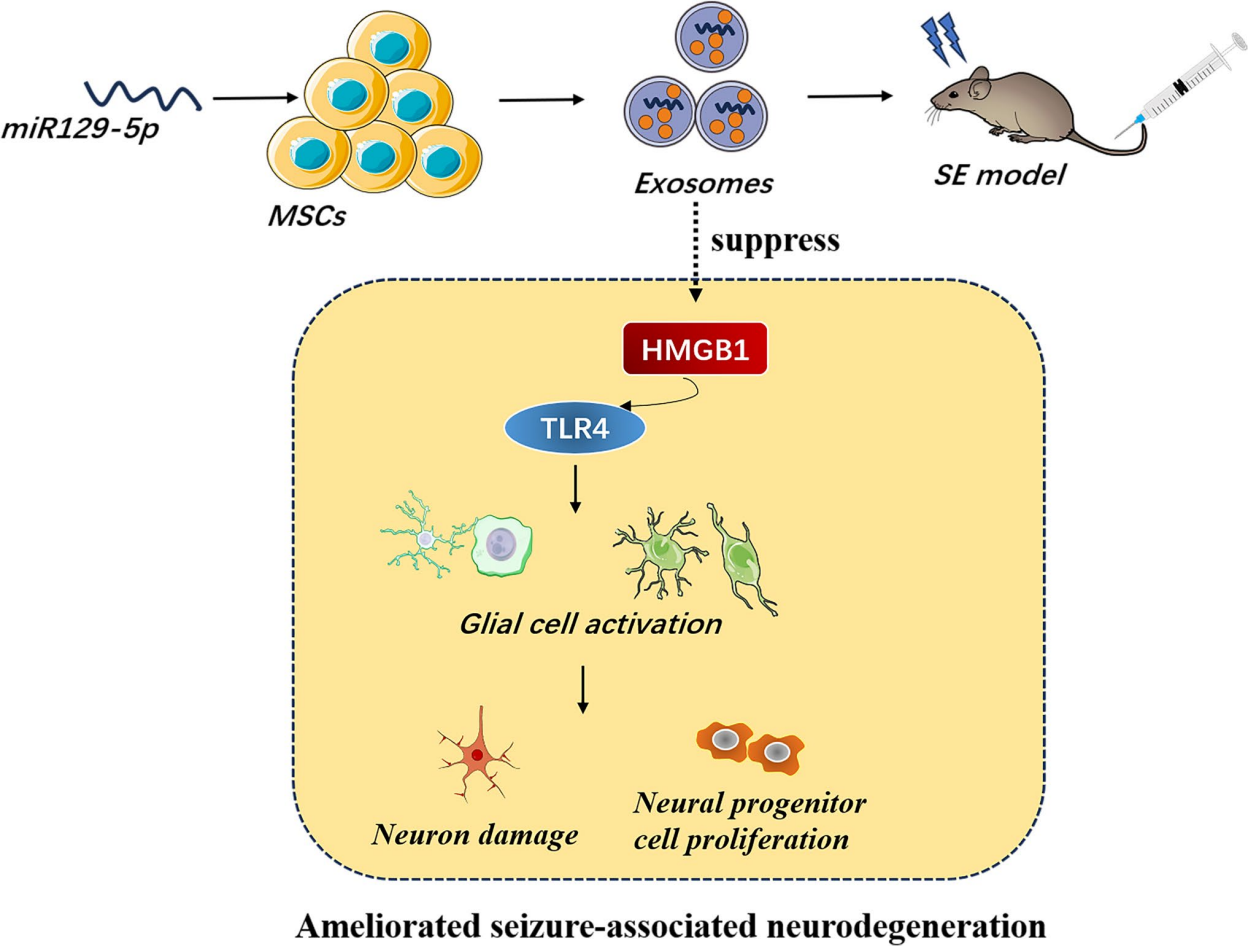

## Introduction

Epilepsy is characterized by spontaneous and recurrent episodes of neuronal hyperactivity (seizures) that can lead to progressive neurological damage [1, 2]. Epileptic seizures are usually terminated by multiple endogenous homeostatic mechanisms, but these can be disrupted by chronic seizure activity, leading to potentially fatal SE, defined clinically by seizures lasting longer than 5 min or by more than one seizure within 5 min and leading to abnormally, prolonged seizures [3, 4]. The sustained seizure activity of SE can induce acute excitotoxic neuronal injury or death, neuroinflammation and secondary neuronal injury, and abnormal neurogenesis, ultimately leading to functional deficits and greater risk of future SE [3, 5]. Moreover, currently available antiepileptic drugs are only partially effective on reducing seizures and preventing SE, consequently, epilepsy remains a fatal neurological disorder [6].

Neuroinflammation is a major pathomechanism contributing to epilepsy progression and associated neurological dysfunction [7]. Seizures induce an inflammatory cytokine storm characterized by activation of glial cells and release of pro-inflammatory factors such as interleukin (IL)-1β, IL-6, and tumor necrosis factor (TNF)-α [8–10]. These neuroinflammatory responses induce a progressive self-sustaining cycle of neuronal hyperexcitability, neuronal injury, and aberrant neural plasticity that ultimately reduces seizure threshold in susceptible regions such as the hippocampus [11]. Accordingly, controlling inflammatory signaling may serve as an effective means to prevent epileptic seizures. Indeed, anti-inflammatory drugs have demonstrated anti-epileptic effects in animal models and human patients [12, 13]. Moreover, these agents can prevent neurodegeneration secondary to epilepsy-induced neuroinflammation [14]. However, anti-inflammatory drugs have a myriad of deleterious side effects and suppress inflammatory signaling pathways for a relatively limited period of time. Thus, long-lasting strategies targeting epilepsy-associated neuroinflammatory pathways are required.

MicroRNAs (miRNAs) are endogenous non-coding RNAs that regulate gene expression primarily by binding to the 3′-UTR of complementary (target) mRNAs, thereby influencing a myriad of biological processes including cell proliferation and differentiation [15, 16]. Dysregulation of miRNAs is implicated in the pathogenesis of multiple central nervous system (CNS) diseases including epilepsy [17]. Differential expression of numerous miRNAs has been detected in blood and brain samples of epileptic patients. However, free miRNAs are easily degraded in body fluids and by intracellular lysosomal pathways, limiting therapeutic applicability. Alternatively, it is possible to facilitate the transmission of miRNAs into target cells via exosomes, small (30–100 nm) vesicles containing proteins, lipids, and miRNAs secreted by most cell types [18]. These structures can also pass through the blood–brain barrier (BBB), allowing delivery into the brain by systemic injection, while the double-layer lipid membrane both protects miRNAs from breakdown or chemical modification and promotes intracellular delivery via membrane fusion. The therapeutic potential of miRNA-loaded exosomes has been widely examined, including mesenchymal stem cell (MSC)-derived exosomes loaded with miR-133b in a rat model of intracerebral hemorrhage [19].

The miR-129 family member miR-129-5p is widely reported to suppress tumor formation [20] by negatively regulating the expression of high mobility group box 1 protein (HMGB1) [21], a multifunctional regulator of gene expression, including genes associated with neuroinflammation and oxidative stress. Moreover, HMGB1 is released by immune cells or glia cells and neurons in the CNS [22], and release is upregulated in the brain of epileptic patients and epileptic animal models [23–25], strongly implicating this protein in the pathophysiology of epilepsy.

Therefore, we can suggest a hypothesis that miR129-5p may decrease the release of HMGB1 and inflammatory factors in the epileptic brain, mitigating SE-induced neurological damage. We aim to explore the effect of miR129-5p-loaded exosomes on KA-induced SE and indicate the underlying mechanism.

## Materials and methods

### MiR129-5p transfection of MSCs

Mesenchymal stromal cells (MSCs) were isolated and collected from 6 to 8-week-old male C57BL/6 J mice as described [26] and cultured in Dulbecco’s modified Eagle’s medium (DMEM, Gibco, USA) supplemented with 10% fetal bovine serum (FBS, Gibco) and 1% penicillin/streptomycin at 37 °C under a 5% CO_2_ 95% air atmosphere. Cultures were fed fresh DMEM 48 h after isolation and once every 2 days thereafter. At 90% confluence, the medium was replaced with DMEM plus exosome-free FBS and MSCs were transfected with miR126-5p mimic or mimic NC (Ruibo Biotechnology, Guangzhou, China) for 72 h using the riboFECT CP Transfection Kit according to the manufacturer’s instructions.

### MSC-exo extraction and identification

After transfection, the medium was collected and exosomes isolated using Exosome Isolation Reagent (Ruibo Biotechnology) according to the manufacturer’s instructions. The collected exosomes were identified by Western blot detection of markers CD9, CD63, and CD81, and by analyzing shape and size by electron microscopy. Subsequently, the expression levels of miR129-5p and exo-con were confirmed by RT-PCR.

### Animals

All animal experiments were approved by the Ethics Committee on Experimental Animals of Xuzhou Medical University (Approval number 202203A020). Adult C57/BL6J male mice (8 weeks of age) weighing 20–25 g were provided by the Experimental Animal Center of Xeuzhou Medical University, China, and housed in a specific pathogen-free (SPF) environment at 22 ± 2 °C under a 12-h/12-h light/dark cycle.

### Kainic acid-induced SE and exosome injection

Male mice were randomly divided into four groups (n = 5/group): CON, KA, KA + Exo-con, and KA + Exo-miR129-5p. Mice allocated to the 3 KA groups were administered 25 mg/kg KA (Sigma-Aldrich, USA) by intraperitoneal injection while CON group mice were injected with equal volume saline. Seizures were classified according to the Racine scale [27] as follows: grade 0, no response; grade 1, facial myoclonus; grade 2, head nodding; grade 3, forelimb clonus; grade 4, rearing and severe forelimb clonus; grade 5, rearing, falling, and severe forelimb clonus. Only mice exhibiting stage 3–5 seizures were considered eligible for continued treatment, other mice were excluded from experiments. After SE, mice exhibited a latent seizure-free phase. Diazepam (10 mg/kg, i.p.) was administered 1 h after KA to terminate convulsions. Mice of the KA + Exo-con and KA + Exo-miR129-5p groups were administered exo-con and exo-miR129-5p (100 μg in PBS), respectively, by tail vein injection 24 h after KA injection.

### Western blotting

Hippocampal tissues were extracted from mice following the indicated treatment protocol (Sect. 4.4), incubated in RIPA lysis buffer (Beyotime, Haimen, China) supplemented with the protease inhibitor PMSF, homogenized by homogenizer and centrifuged at 12000 rpm for 15 min at 4 °C. Subsequently, the supernatant was collected and the protein concentration determined using a BCA protein assay kit (Beyotime). Lysate samples were mixed with 5 × (v/v) loading buffer and boiled in a water bath for denaturation. The denatured protein samples (100 μg per gel lane) were separated by sodium dodecyl sulfate polyacrylamide gel electrophoresis (SDS-PAGE) and then transferred to nitrocellulose membranes. Membranes were blocked with 5% non-fat milk, incubated with rabbit anti-HMGB1 monoclonal antibody (1:1000, abcam, UK) and rabbit anti-TLR4 polyclonal antibody (1:1000, abcam, UK) overnight at 4 ℃, rinsed with tris-buffered saline plus Tween-20 (TBST), incubated with Dylight 800 goat anti-rabbit IgG at room temperature (r/t) for 1.5 h, and rinsed again in TBST. Immunolabeling was recorded using an Odyssey scanner (LI-COR, USA) and quantified using ImageJ (NIH, Bethesda, MD, USA).

### Real-time PCR

Total RNA was extracted from mouse hippocampus using the RNA Easy Fast Tissue/Cell Kit (Tiangen, Beijing, China) according to the manufacturer’s instructions and reverse transcribed to cDNA using the PrimeScript RT reagent kit (Takara, Dalian, China) following the manufacturers’ instructions. The total reaction system contained 2 μL cDNA, 0.4 μL forward primer, 0.4 μL reverse primer, and 10 μL SYBR Premix ExTaq II (Takara, Dalian, China). The reaction conditions for measuring miR129-59 expression were 35 cycles of denaturation at 95 °C for 15 s, annealing at 60 °C for 1 min, and termination at 95 °C for 15 s. The primers for RT-qPCR were as follows: F: 5′-ACCCAGTGCGATTTGTCA-3′, R: 5′-ACTGTACTGGAAGATGGACC-3′.

### Enzyme-linked immunosorbent assays (ELISAs)

Hippocampal tissues were dissociated and collected 48 h after KA induction. The hippocampal concentrations of IL-1β, IL-6, and TNF-α were measured using ELISA kits (Jianglaibio, Shanghai, China) according to the manufacturer’s protocol. In brief, hippocampal lysates were incubated with reaction buffer with five holes, followed by incubation for 2.5 h at r/t. Terminating the reaction after 30 min of substrate coloration. Absorbance was measured using a Synergy2 microplate reader (BIO-TEK) and converted to pg per mg of total protein.

### Nissl and FJB staining

Mice were perfused through the heart with 4% polyformaldehyde 48 h after KA induction and brain tissues isolated, paraffin-embedded, and sectioned at 5 μm thickness. Sections were dehydrated, treated with Nissl staining solution at 37 °C for 10 min, immersed in 70% alcohol for 5 s, mounted with neutral balsam, and imaged using an IX71 microscope (Olympus, Tokyo, Japan). The number of Nissl-positive neurons in the hippocampal CA3 area was calculated using ImageJ software.

For Fluoro-Jade B (FJB) staining, tissue sections were incubated in a 1:50–1:100 mixture of 0.1% glacial acetic acid and FJB at r/t for 20 min, rinsed with pure water, counterstained with DAPI, rinsed with PBS, mounted under coverslips, and imaged using an IX71 fluorescence microscope (Olympus, Tokyo, Japan).

### Immunofluorescence staining

After fixation (Sect. 4.8), brain tissues were dehydrated in 30% sucrose for 72 h, sliced in the coronal plane at 30 μm thickness using a freezing microtome, and processed for BrdU staining. First, genomic DNA in slices was denatured by treatment with 2 Ν HCl for 30 min at 37 °C. Slices were then neutralized by immersed in 0.1 M sodium borate for 10 min, and then rinsed with PBS. The tissues were permeabilized with 0.3% Triton X-100 PBS solution, blocked with 10% goat serum for 60 min at r/t, and incubated with mouse anti-IBA-1 monoclonal antibody (1:1000, Abcam, UK), rabbit anti-GFAP polyclonal antibody (1:1000, Abcam), and mouse anti-BrdU monoclonal antibody (1:1000, Abcam) overnight at 4 °C. Slices were subsequently rinsed with PBS, incubated with FITC-conjugated goat anti-rabbit IgG Alexa Fluor 594 conjugate (1:500, Abcam, UK) and goat anti-mouse IgG Alexa Fluor 488 conjugate (1:500, Abcam) for 2 h at r/t, counterstained and mounted with DAPI Fluoromount-G (Beyotime), and imaged using an IX71 fluorescence microscope (Olympus, Tokyo, Japan).

### Image capture

All the immunostaining images were captured using an IX71 fluorescence microscope (Olympus, Tokyo, Japan) under different magnifications. The images of IBA-1, GFAP fluorescent staining, Nissl staining and FJB staining were captured at 20 × objective, while BrdU staining image were captured under 10 × objective.

### Statistical analysis

Statistical Package for Social Sciences (SPSS) software version 20 was used for the statistical analysis. The datasets were examined for normality using a Q-Q plot test. Two groups were compared by Student’s T-test and three or more groups by one- or two-way ANOVA followed by Tukey’s post hoc tests. All data are presented as the mean ± SEM. A p < 0.05 was considered statistically significant for all tests.

## Results

### Elevated HMGB1 expression and reduced miR129-5p expression in the hippocampus of SE model mice

**Fig. 1:**
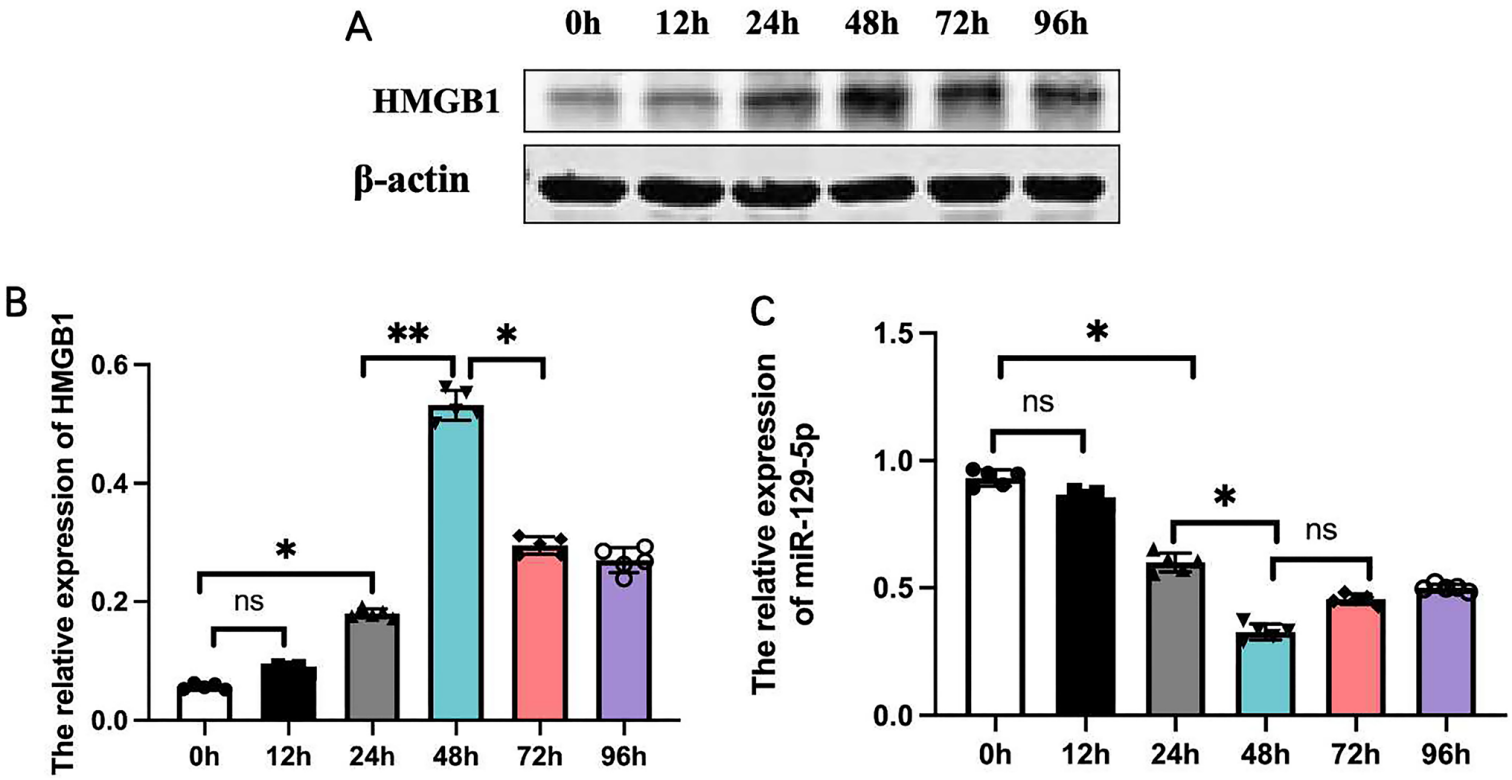
Downregulation of miR129-5p expression and concomitant upregulation of HMGB1 protein in the mouse hippocampus following KA-induced SE. **A** Hippocampal HMGB1 expression was detected at 12, 24, 48, 72, and 96 h after KA injection by western blot. **B** Changes in HMGB1 expression over time as measured by relative optical density (ROD). Results are expressed as the mean ± SEM. **C** MiR129-5p expression in hippocampus at 12, 24, 48, 72, and 96 h after KA injection measured by qPCR. *n* = *5* mice per group **P < 0.01, *P < *0.05*

Western blotting of hippocampal tissue extract from the four mouse groups 12, 24, 48, 72, and 96 h after KA injection revealed that SE significantly enhanced HMGB1 expression, with upregulation peaking at 48 h (Fig. 1A, B) conducted via ANOVA (F = 626.32, df = 24, p = 0.0001). In contrast, qPCR revealed that miR129-5p expression was downregulated by SE (Fig. 1C), with lowest expression at 48 h after KA injection, conducted via ANOVA (F = 373.61, df = 24, p = 0.0001). These findings suggest that miR129-5p serves to inhibit HMGB1 expression in the hippocampus and that preventing miR129-5p downregulation may suppress the expression of HMGB1, a potential mediator of SE-induced neuroinflammation and neuronal damage. Accordingly, in the subsequent experiment, we collected samples for detection at 48 h after SE.

### Exo-miR129-5p injection inhibited HMGB1/TLR4 expression in the hippocampus following SE

**Fig. 2:**
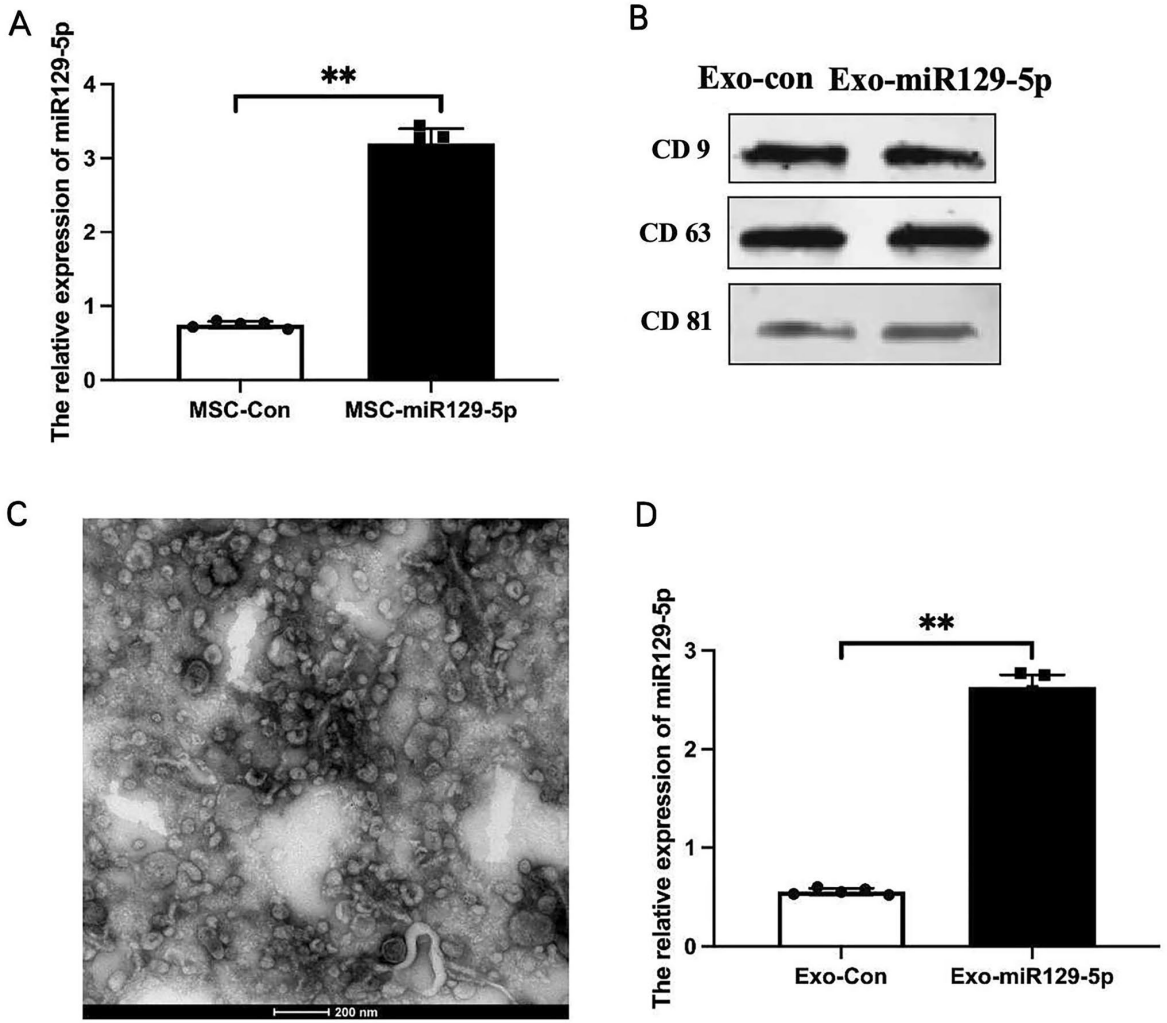
Extraction and identification of miR129-5p-loaded exosomes. **A** Detection of miR129-5p overexpression in transfected MSCs by qPCR. **B** Expression levels of exosomal protein markers CD9, CD63, and CD81 measured by western blotting. **C** Extracellular vesicle size determination by electron microscopy. The size distribution is consistent with that of exosomes **D** Relative expression levels of miR129-5p in MSC-derived exosomes detected by qPCR. Expression was enriched in exosomes from MSCs transfected with miR129-5p mimic compared to MSCs transfected with NC miRNA. *n* = *5* independently transfected cultures per group. **P < *0.01*

Mesenchymal stem cells were transfected with miR129-5p mimic or negative control miRNA, and successful overexpression confirmed by RT-PCR (Fig. 2A).

**Fig. 3:**
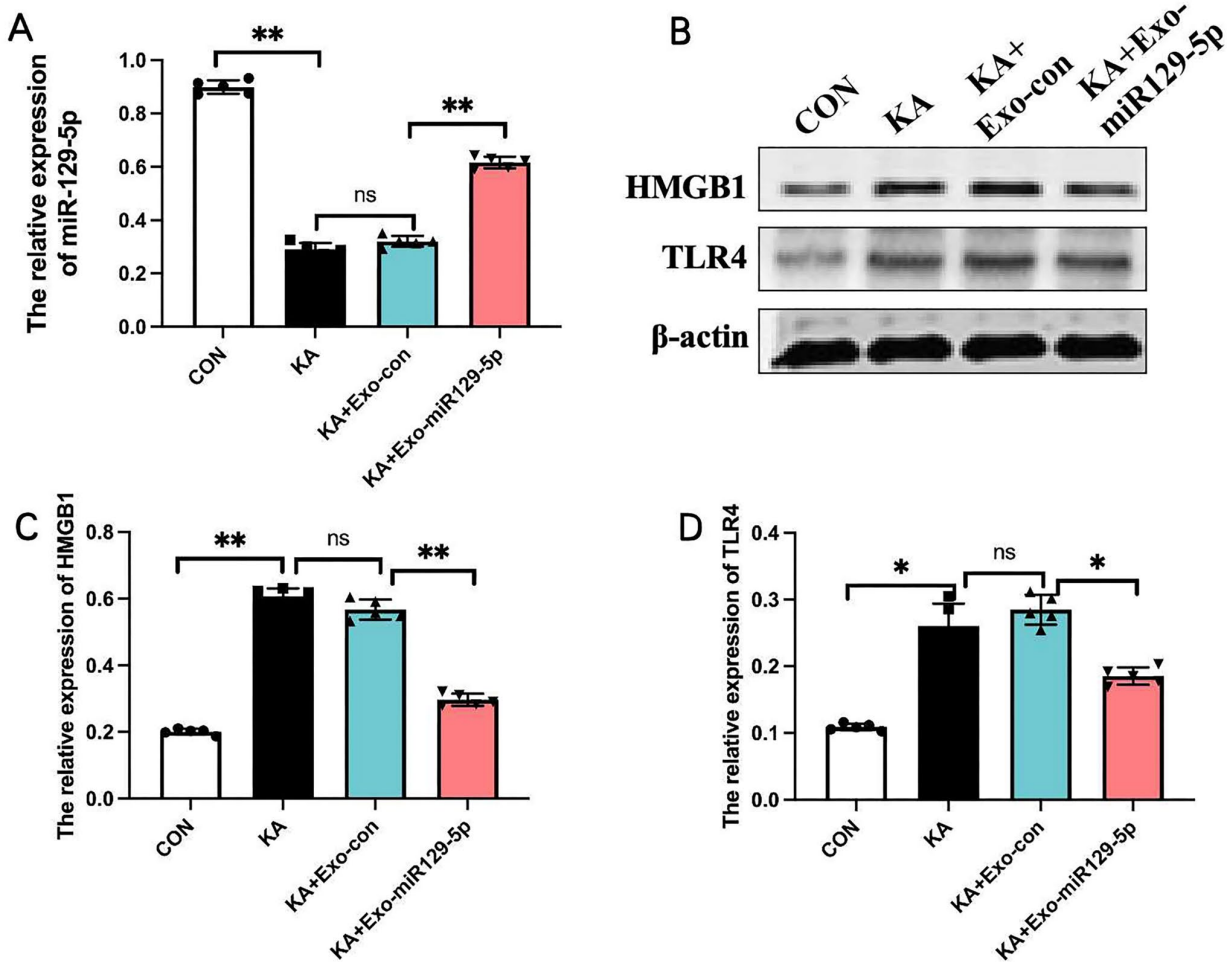
Injection of exo-miR129-5p reversed the SE-induced increase in hippocampal HMGB1/TLR4 expression. **A** Relative expression levels of miR129-5p in the hippocampus of CON, KA, KA + Exo-con, and KA + Exo-miR129-5p group mice detected by qPCR. **B** Relative protein expression levels of HMGB1 and TLR4 in the hippocampus of CON, KA, KA + Exo-con, and KA + Exo-miR129-5p group mice determined by western blot. **C**, **D** Hippocampal HMGB1 and TLR4 expression levels determined by relative optical density (ROD) measures following immunostaining *n* = *5* mice per group, **P < 0.01, *P < *0.05*

The relative expression of miR129-5p was significantly higher in MSC-miR129-5p group (0.75 ± 0.04) than in MSC-Con group (3.20 ± 0.19), conducted via T-test (t = − 27.071, 95% CI = − 2.70 to − 2.21, p = 0.0002). Extracellular vesicles were then extracted from the medium of transfected MSCs after 72 h and exosomal identity confirmed by detection of the markers CD9, CD63, and CD81 (Fig. 2B). Further, electron microscopy revealed that these vesicles were within the range of typical exosomes (100 ± 50 nm) (Fig. 2C). Injection of Exo-miR129-5p (0.61 ± 0.02) into the tail vein upregulated miR129-5p expression in the hippocampus compared to Exo-con injection (0.32 ± 0.02) (Fig. 3A), which was statisticly analyzed with T-test. Moreover, this upregulation was associated with downregulation of hippocampal HMGB1 and TLR4 expression levels as determined by western blot (Fig. 3B). Thus, systemic injection of exo-miR129-5p can enhance hippocampal expression and subsequently interfere with the expression of target genes (encoding HMGB1) and downstream genes (TLR4) (Fig. 3C, D). T-test analysis showed the expression of HMGB1 in KA + Exo-miR129-5p group (0.30 ± 0.02) was decreased than KA + Exo-con group (0.57 ± 0.03), (t = 17.22, 95% CI = − 0.23 to − 0.31, p = 0.007). The expression of TLR4 in KA + Exo-miR129-5p group (0.25 ± 0.06) was downregulated than KA + Exo-con group (0.61 ± 0.01), (t = 2.39, 95% CI = − 0.002 to − 0.121, p = 0.04).

### Exo-miR129-5p injection suppressed hippocampal inflammation following SE

**Fig. 4:**
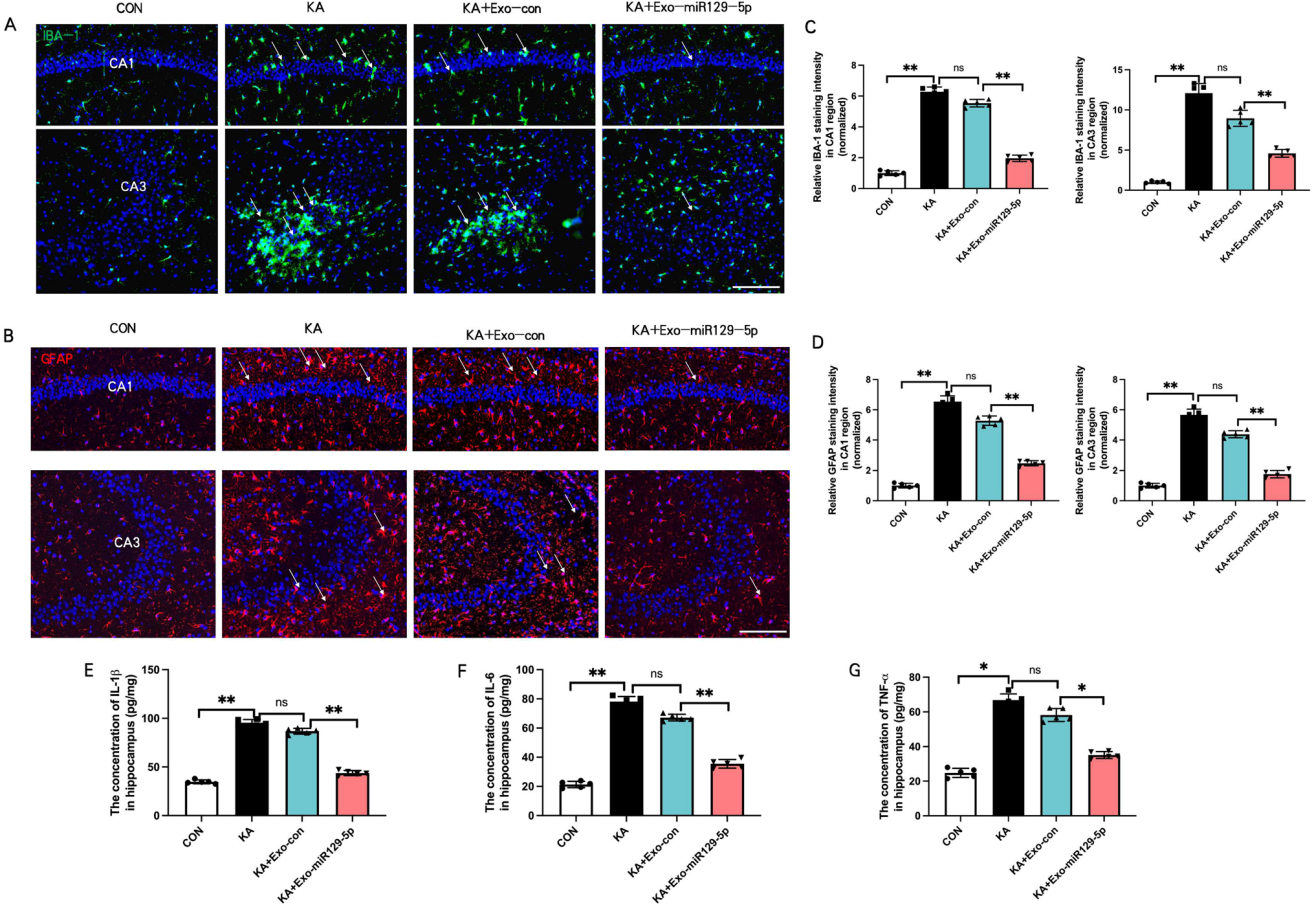
Injection of exo-miR129-5p suppressed SE-induced hippocampal inflammation. **A**, **B** Immunofluorescence staining of the reactive microglial marker IBA-1 and reactive astrocyte marker GFAP in CA1 and CA3 regions of hippocampus. **C**, **D** Statistical histogram of IBA-1 and GFAP staining intensity. **E**, **F**, **G** Concentrations of inflammatory factors IL-1β, IL-6, and TNF-α in the hippocampus following SE induction determined by ELISA. *n* = *5* mice per group, **P < 0.01, *P < *0.05*

Kainic acid-induced SE is associated with neuroinflammation driven in part by the reactive transformation (immune activation) of glial cells. Injection of exo-miR129-5p reduced the reactive transformation of microglia and astrocytes in hippocampal CA1 and CA3 as evidenced by lower IBA-1 + microglia and GFAP + astroglial staining intensity compared to KA + exo-con group (Fig. 4A, B). Statistic analysis conducted with T-test analysis showed IBA-1 staining intensity in CA1 (1.96 ± 0.20) and CA3 (4.60 ± 0.47) in KA + Exo-miR129-5p group were significantly reduced than that (5.54 ± 0.24; 8.96 ± 0.99) in KA + Exo-con group (t = 25.19, 95% CI = 3.26 to 3.91, p = 0.006; t = 8.80, 95% CI = 3.22 to 5.50, p = 0.003) (Fig. 4C). Similarly, GFAP staining intensity in CA1 (2.46 ± 0.17) and CA3 (1.76 ± 0.24) in KA + Exo-miR129-5p group were significantly reduced than that (5.28 ± 0.31; 4.38 ± 0.23) in KA + Exo-con group (t = 17.84, 95% CI = 2.46 to 3.18, p = 0.008; t = 17.28, 95% CI = 2.27 to 2.97, p = 0.005) (Fig. 4 D). In addition, exo-miR129-5p reduced hippocampal concentrations of the pro-inflammatory factors IL-1β, IL-6, and TNF-α compared to KA and KA-exo-con group mice as determined by ELISA (Fig. 4E–G). The concentrations of IL-1β, IL-6, and TNF-α were significantly decreased in KA + Exo-miR129-5p group (4.37 ± 2.54; 3.55 ± 2.96; 35.16 ± 1.99) than in KA + Exo-con group (8.67 ± 2.97; 6.71 ± 2.28; 58.24 ± 3.74) analyzed via T-test analysis (t = 24.61, 95% CI = 38.98 to 47.05, p = 0.005; t = 18.91, 95% CI = 27.78 to 35.49, p = 0.007; t = 12.18, 95% CI = 18.46 to 27.70, p = 0.04).

### Exo-miR129-5p injection suppressed hippocampal neuronal damage following SE

**Fig. 5:**
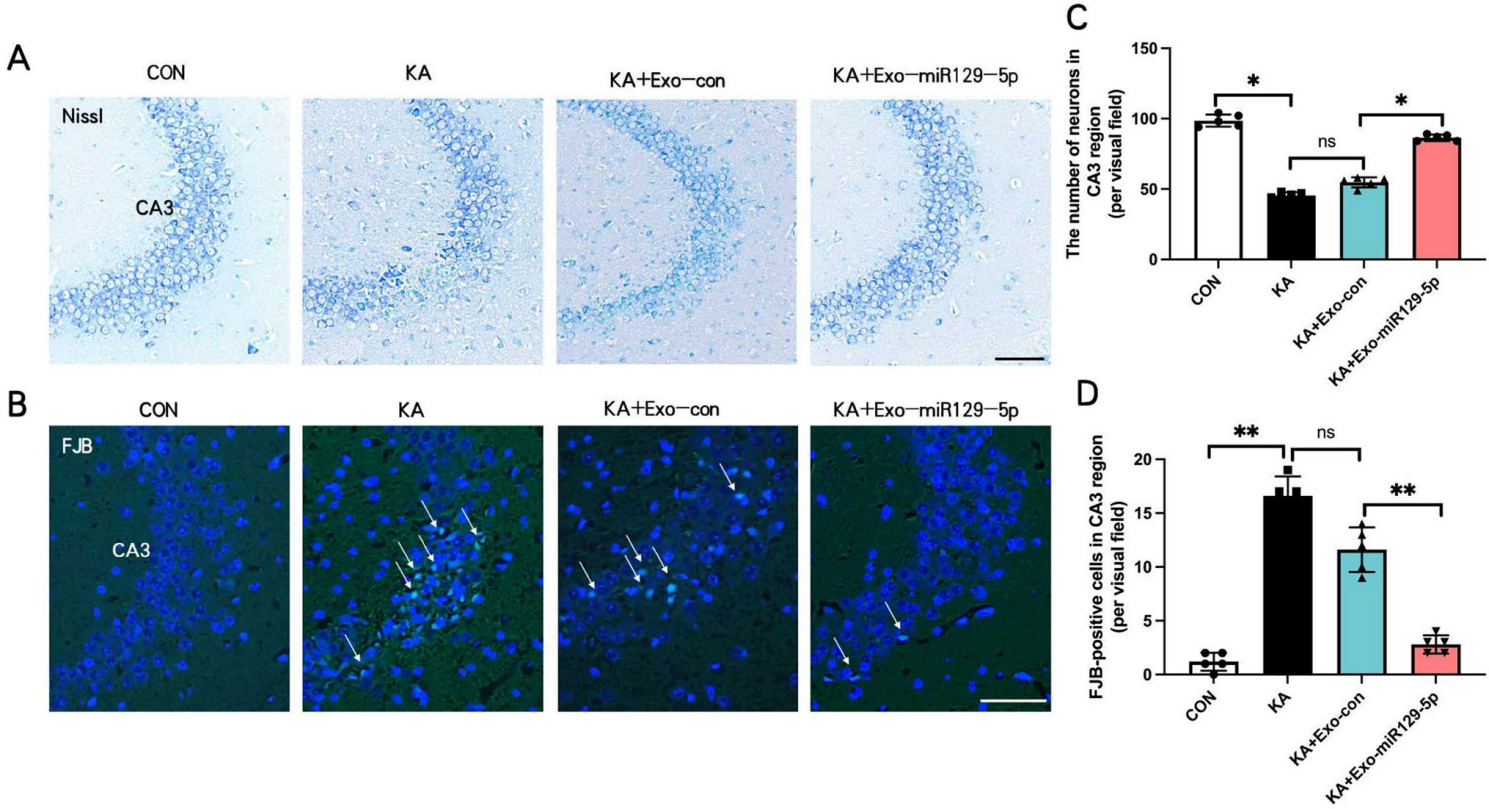
Injection of exo-miR129-5p prevented hippocampal neuronal damage following SE. **A**, **B** Representative images of Nissl staining and FJB staining in the hippocampal CA3 region. **C**, **D** Statistical histogram of neuronal number and FJB-positive cells in CA3. *n* = *5* mice per group, **P < 0.01, *P < *0.05*

Nissl staining revealed substantial neuronal loss in the hippocampal CA3 region of KA and KA + exo-con group mice, which was reversed by exo-miR129-5p injection (Fig. 5A, C). The counting of neurons of CA3 region in KA + Exo-miR129-5p group (86.4 ± 2.30) was remarkedly increased than in KA + Exo-con group (54.8 ± 3.56) analyzed via T-test analysis (t = − 16.66, 95% CI = − 35.97 to − 27.22, p = 0.02), while that in CA1 region was no significant diffference in CON group and in KA group (p = 0.08). Further, SE in KA and KA + exo-con group mice produced a marked rise in FJB + neuron count, which again was reversed by exo-miR129-5p injection (Fig. 5B, D). The counting of FJB-positive cells of CA3 region in KA + Exo-miR129-5p group (2.8 ± 0.83) was remarkedly decreased than in KA + Exo-con group (11.6 ± 2.07) analyzed via T-test analysis (t = 8.80, 95% CI = 6.27 to11.33, p = 0.001). Thus, exo-miR129-5p injection alleviated neuronal loss and degeneration in the CA3 region of KA-induced SE model mice, possibly by suppressing neuroinflammation.

### Exo-miR129-5p injection decreased neural progenitor cell proliferation in the DG following SE

**Fig. 6:**
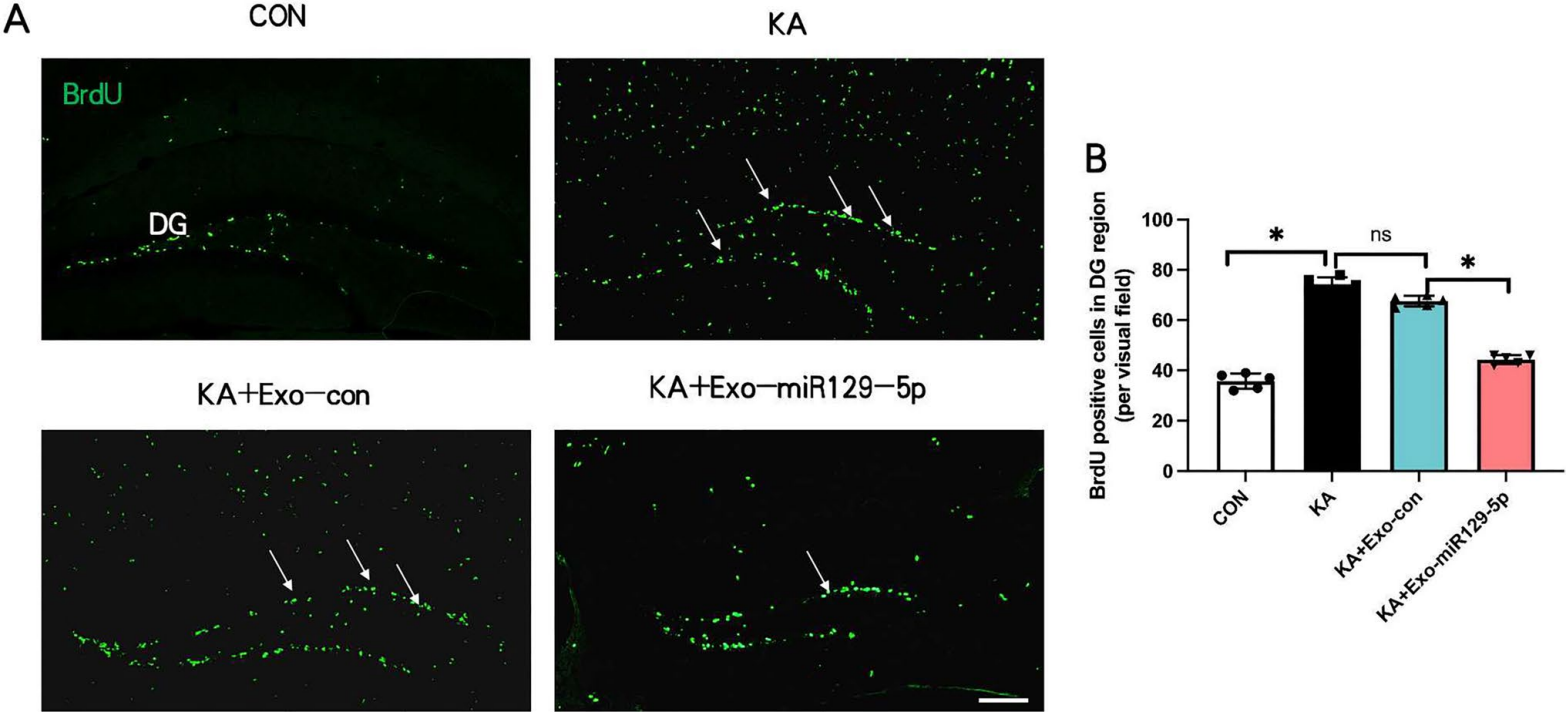
Injection of exo-miR129-5p blocked SE-induced neurogenesis in the dentate gyrus (DG). **A** Representative images of BrdU immunofluorescence staining in the DG. **B** Number of BrdU-positive cells in the DG. *n* = *5* mice per group, *P < *0.05*

Finally, BrdU staining revealed that KA-induced SE markedly enhanced the rate of neural progenitor cell proliferation (Fig. 6A, B), and this response that was reduced by exo-miR129-5p treatment. The number of BrdU-positive cells in DG in KA + Exo-miR129-5p group (4.44 ± 1.81) was significantly reduced than in KA + Exo-con group (6.76 ± 2.07) analyzed via T-test analysis (t = 18.82, 95% CI = 20.36 to 26.04, p = 0.02).

## Discussion

Neuroinflammation is a key pathophysiological characteristic of the epileptic brain, and in some cases, inflammation contributes to the progression and recurrence of epilepsy. Annamaria et al. proposed that targeting neuroinflammation-related pathways may be an effective anti-epileptogenic and disease-modifying strategy [8]. Indeed, anti-inflammatory drugs have demonstrated therapeutic efficacy on drug-resistant seizures [8]. Moreover, anti-inflammatory interventions are reported to improve the disease course, reduce seizure frequency, and promote neuroprotection in animal models of epilepsy [28]. Here we show that miRNA-mediated downregulation of the inflammatory signaling factor HMGB1 can suppress SE-induced hippocampal inflammation and neuronal death.

Consistent with our results, HMGB1 was reported to be upregulated in both animal models of epilepsy and epileptic patients [29, 30], suggesting miRNA-mediated suppression of HMGB1 expression as a potential therapeutic strategy for SE-associated neuroinflammation and neurodegeneration. However, delivery of exogenous miRNAs is hampered by the instability of these molecules in the extracellular environment. Exosomes are important conduits for cell-to-cell communication by transporting proteins, miRNA, circRNA, lncRNA, and other components [26], and numerous studies support the therapeutic potential of exosomes loaded with various protective factors. Mesenchymal stromal cells can be conveniently isolated from various tissues and manipulated to release microsomes enriched in therapeutic factors. Moreover, MSCs naturally release a variety of neuroprotective factors [31]. Accordingly, MSC-derived exosomes have garnered intense interest as sources for therapeutic exosomes. Here, we extracted exosomes from MSCs transfected with miR129-5p mimic and found that these exosomes were enriched in miR129-5p. Moreover, systemic injection of these exosomes increased miR129-5p expression in the hippocampus, supporting the feasibility of therapeutic application. Consistent with this notion, Li and colleagues reported that increased miR129-5p expression ameliorated neuroinflammation after ischemia–reperfusion by inhibiting HMGB1 and the TLR3-cytokine pathway [32]. The current findings expand the spectrum of potential therapeutic applications to SE-associated neuroinflammation through downregulation of hippocampal HMGB1 and TLR4.

High mobility group box 1 protein is released from neurons, astrocytes, and microglia under pathological conditions and activates the TLR4 signaling pathway, triggering neuroinflammation. Status epilepticus results in activation of microglia and astrocytes and ensuing release of inflammatory factors [10, 33, 34]. Immunostaining for markers of activated microglia and astrocytes revealed that the SE-induced increases in expression were reversed by exo-miR-129-5p concomitant with reductions in IL-1β, IL-6, and TNF-α concentrations. Collectively, these results strongly suggest that miR129-5p suppresses SE-induced neurodegeneration by inhibiting HMGB1/TLR4 and downstream neuroinflammatory pathways.

Chronic neuroinflammation may damage neurons by promoting hyperexcitability, initiating a perpetuating cycle of increased seizure activity, neuroinflammation, and neuronal damage [35, 36]. Neuroinflammation can in turn trigger hippocampal neurogenesis [10]. Here, we report that systemic exo-miR129-5p administration reduced neuronal damage in CA3 region as evidenced by Nissl and FJB staining. Here, we observed no significant difference of neurons in CA1 region after SE induction, which may be due to differences in molding methods or drug dosage. In addition exo-miR129-5p reduced neurogenesis in the DG as evidenced by BrdU labeling. There is compelling evidence that proliferation of progenitor cell contributes to the changes in circuit structure underlying further seizure activity [11, 37]. Accordingly, this decrease in neurogenesis may prevent the progressive increase in seizure frequency and severity observed among many TLE patients. These findings provide evidence that miR129-5p-loaded exosomes may be a promising strategy to relieve pathological damage in epileptic brain. Additionally, the small sample size can be a limitation in our study, we will expand the sample size for further in-depth research. In this study, we mainly focus on neuroinflammation, and further exploration is needed to determine whether there is inhibitory effect on epileptic discharge and neuron activities.

## Conclusion

In conclusion, we demonstrate miR129-5p expression is reduced in the hippocampus of SE model mice and that prevention of this decrease by systemic administration of MSC-derived miR129-5p-enriched exosomes can prevent SE-induced neurological damage by suppressing the pro-inflammatory HMGB1/TLR4 signaling axis.

## Data Availability

The data used to support the findings of this study are available from the corresponding author upon request.

## Abbreviations

BBB: Blood–brain barrier
CNS: Central nervous system
DG: Dentate gyrus
HMGB1: High mobility group box 1
KA: Kainic acid
MSC: Mesenchymal stem cell
SE: Status epilepticus

## References

[1] Devinsky O, Vezzani A, O’Brien TJ, Jette N, Scheffer IE, de Curtis M, Perucca P (2018) Epilepsy Nat Rev Dis Primers 3(4):18024 10.1038/nrdp.2018.24 29722352

[2] Thijs RD, Surges R, O’Brien TJ, Sander JW (2019) Epilepsy in adults. Lancet 393:689–701 30686584 10.1016/S0140-6736(18)32596-0

[3] Andres-Mach M, Szewczyk A, Zagaja M, Szala-Rycaj J, Lemieszek MK, Maj M, Abram M, Kaminski K (2021) Preclinical assessment of a new hybrid compound C11 efficacy on neurogenesis and cognitive functions after pilocarpine induced status epilepticus in mice int. J Mol Sci 22(6):3240 10.3390/ijms22063240 PMC8004689 33810180

[4] Trinka E, Cock H, Hesdorffer D, Rossetti AO, Scheffer IE, Shinnar S, Shorvon S, Lowenstein DH (2015) A definition and classification of status epilepticus—report of the ILAE task force on classification of status epilepticus. Epilepsia 56:1515–1523 26336950 10.1111/epi.13121

[5] Castro OW, Upadhya D, Kodali M, Shetty AK (2017) Resveratrol for easing status epilepticus induced brain injury, inflammation, epileptogenesis, and cognitive and memory dysfunction—are we there yet? Front Neurol 8:603 29180982 10.3389/fneur.2017.00603 PMC5694141

[6] Hu H, Zhu T, Gong L, Zhao Y, Shao Y, Li S, Sun Z, Ling Y, Tao Y, Ying Y, Lan C, Xie Y, Jiang P (2020) Transient receptor potential melastatin 2 contributes to neuroinflammation and negatively regulates cognitive outcomes in a pilocarpineinduced mouse model of epilepsy. Int Immunopharmacol 87:106824 32731181 10.1016/j.intimp.2020.106824

[7] Vezzani A, Viviani B (2015) Neuromodulatory properties of inflammatory cytokines and their impact on neuronal excitability. Neuropharmacology 96:70–82 25445483 10.1016/j.neuropharm.2014.10.027

[8] Vezzani A, Balosso S, Ravizza T (2019) Neuroinflammatory pathways as treatment targets and biomarkers in epilepsy. Nat Rev Neurol 15(8):459–472 31263255 10.1038/s41582-019-0217-x

[9] Kaneko KI, Irie S, Mawatari A, Igesaka A, Hu D, Nakaoka T, Hayashinaka E, Wada Y, Doi H, Watanabe Y, Cui Y (2022) [(18)F]DPA-714 PET imaging for the quantitative evaluation of early spatiotemporal changes of neuroinflammation in rat brain following status epilepticus. Eur J Nucl Med Mol Imaging 49:2265–2275 35157105 10.1007/s00259-022-05719-7

[10] Hu AK, Yuan HH, Qin Y, Zhu YH, Zhang LZ, Chen QA, Wu LL (2022) Lipopolysaccharide (LPS) increases susceptibility to epilepsy via interleukin-1 type 1 receptor signaling. Brain Res 15:148052 10.1016/j.brainres.2022.148052 35970265

[11] Zhu X, Yao Y, Yang J, Ge Q, Niu D, Liu X, Zhang C, Gan G, Zhang A, Yao H (2020) Seizure-induced neuroinflammation contributes to ectopic neurogenesis and aggressive behavior in pilocarpine-induced status epilepticus mice. Neuropharmacology 170:108044 32179291 10.1016/j.neuropharm.2020.108044

[12] DeSena AD, Do T, Schulert GS (2018) Systemic autoinflammation with intractable epilepsy managed with interleukin-1 blockade. J Neuroinflamm 15(1):38 10.1186/s12974-018-1063-2 PMC5807745 29426321

[13] Zhang YY, Wang ZY, Wang RR, Xia L, Cai YY, Tong FC, Gao YQ, Ding J, Wang X (2022) Conditional knockout of ASK1 in microglia/macrophages attenuates epileptic seizures and long-term neurobehavioural comorbidities by modulating the inflammatory responses of microglia/macrophages. J Neuroinflamm 19(1):202 10.1186/s12974-022-02560-5 PMC9361603 35941644

[14] Gimenes AD, Andrade BFD, Pinotti JVP, Oliani SM, Galvis-Alonso OY, Gil CD (2019) Annexin A1-derived peptide Ac2-26 in a pilocarpine-induced status epilepticus model: anti-inflammatory and neuroprotective effects. J Neuroinflamm 12:32 10.1186/s12974-019-1414-7 PMC6371492 30755225

[15] Bartel DP (2018) Metazoan MicroRNAs. Cell 173:20–51 29570994 10.1016/j.cell.2018.03.006 PMC6091663

[16] Batool A, Hill TDM, Nguyen NT, Langa E, Diviney M, Mooney C, Brennan GP, Connolly NMC, Sanz-Rodriguez A, Cavanagh BL, Henshall DC (2020) Altered biogenesis and MicroRNA content of hippocampal exosomes following experimental status epilepticus. Front Neurosci 17:1404 10.3389/fnins.2019.01404 PMC6978807 32009885

[17] Guoping X, Huan C, Chan H, Siheng H, Xue X, Qunying L (2023) The dysregulation of miRNAs in epilepsy and their regulatory role in inflammation and apoptosis. Funct Integr Genomics 23(3):287 37653173 10.1007/s10142-023-01220-y PMC10471759

[18] Wortzel I, Dror S, Kenific CM, Lyden D (2019) Exosome-mediated metastasis: communication from a distance. Dev Cell 49(3):347–360 31063754 10.1016/j.devcel.2019.04.011

[19] Shen HT, Yao XY, Li HY, Li X, Zhang TJ, Sun Q, Ji CY, Chen G (2018) Role of exosomes derived from miR-133b modified MSCs in an experimental rat model of intracerebral hemorrhage. J Mol Neurosci 64(3):421–430 29455449 10.1007/s12031-018-1041-2

[20] Gao B, Wang LJ, Zhang N, Han MM, Zhang YB, Liu HC, Sun DL, Xiao XL, Liu YF (2021) miR-129-5p inhibits clear cell renal cell carcinoma cell proliferation, migration and invasion by targeting SPN. Cancer Cell Int 21(1):263 34001147 10.1186/s12935-021-01820-3 PMC8127191

[21] Liu AH, Wu YT, Wang YP (2017) MicroRNA-129-5p inhibits the development of autoimmune encephalomyelitis-related epilepsy by targeting HMGB1 through the TLR4/NF-kB signaling pathway. Brain Res Bull 132:139–149 28528202 10.1016/j.brainresbull.2017.05.004

[22] Mukhtar I (2020) Inflammatory and immune mechanisms underlying epileptogenesis and epilepsy: from pathogenesis to treatment target. Seizure-Eur J Epilep 82:65–79 10.1016/j.seizure.2020.09.015 33011590

[23] Vezzani A, French J, Bartfai T, Baram TZ (2011) The role of inflammation in epilepsy. Nat Rev Neurol 7:31–40 21135885 10.1038/nrneurol.2010.178 PMC3378051

[24] Walker L, Sills GJ (2012) Inflammation and epilepsy: the foundations for a new therapeutic approach in epilepsy? Epilepsy Curr 12(1):8–12 22368518 10.5698/1535-7511-12.1.8 PMC3280476

[25] Yue C, Xilu C, Ying L (2023) Meta-analysis of HMGB1 levels in the cerebrospinal fluid and serum of patients with epilepsy. Neurol Sci 44(7):2329–2337 36933099 10.1007/s10072-023-06720-0

[26] Pan Q, Kuang X, Cai S, Wang X, Du D, Wang J, Wang Y, Chen Y, Bihl J, Zhao B, Ma X (2020) miR-132-3p priming enhances the effects of mesenchymal stromal cell-derived exosomes on ameliorating brain ischemic injury. Stem Cell Res Ther 11:260 32600449 10.1186/s13287-020-01761-0 PMC7322840

[27] Racine RJ, Gartner JG, Burnham WM (1972) Epileptiform activity and neural plasticity in limbic structures. Brain Res 47:262–268 4641271 10.1016/0006-8993(72)90268-5

[28] Terrone G, Balosso S, Pauletti A, Ravizza T, Vezzani A (2020) Inflammation and reactive oxygen species as disease modifiers in epilepsy. Neuropharmacology 167:107742 31421074 10.1016/j.neuropharm.2019.107742

[29] Terrone G, Frigerio F, Balosso S, Ravizza T, Vezzani, (2019) Inflammation and reactive oxygen species in status epilepticus: biomarkers and implications for therapy. Epilepsy Behav 101:106275 31171434 10.1016/j.yebeh.2019.04.028

[30] Wang KY, Yu GF, Zhang ZY, Huang Q, Dong XQ (2012) Plasma high-mobility group Box 1 levels and prediction of outcome in patients with traumatic brain injury. Clin Chim Acta 413:1737–1741 22789964 10.1016/j.cca.2012.07.002

[31] Wang L, Qing L, Liu H, Liu N, Qiao J, Cui C, He T, Zhao R, Liu F, Yan F, Wang C, Liang K, Guo X, Shen Y, Hou X, Chen L (2017) Mesenchymal stromalvcells ameliorate oxidative stress-induced islet endothelium apoptosis andvfunctional impairment via Wnt4-beta-catenin signaling. Stem Cell Resv Ther 8(1):188 10.1186/s13287-017-0640-0 PMC5557510 28807051

[32] Li XQ, Chen FS, Tan WF, Fang B, Zhang ZL, Ma H (2021) Elevated microRNA-129-5p level ameliorates neuroinflammation and blood-spinal cord barrier damage after ischemia-reperfusion by inhibiting HMGB1 and the TLR3-cytokine pathway. J Neuroinflammation 18(1):308 34963474 10.1186/s12974-021-02345-2 PMC8715607

[33] Zhu X, Liu J, Chen O, Xue J, Huang S, Zhu W, Wang Y (2019) Neuroprotective and anti-inflammatory effects of isoliquiritigenin in kainic acid-induced epileptic rats via the TLR4/MYD88 signaling pathway. Inflammopharmacology 27(6):1143–1153 31037573 10.1007/s10787-019-00592-7

[34] Wu LL, Qin Y, Yuan HH, Zhu YH, Hu AK (2022) Anti-inflammatory and neuroprotective effects of insulin-like growth factor-1 overexpression in pentylenetetrazole (PTZ)-induced mouse model of chronic epilepsy. Brain Res 1785:147881 35283097 10.1016/j.brainres.2022.147881

[35] Wolinski P, Ksiazek WD, Glabinski A (2022) Cytokines and neurodegeneration in epileptogenesis. Brain Sci 12(3):380 35326336 10.3390/brainsci12030380 PMC8945903

[36] Shin HJ, Lee JY, Son E, Lee DH, Kim HJ, Kang SS, Cho GJ, Choi WS, Roh GS (2007) Curcumin attenuates the kainic acid-induced hippocampal cell death in the mice. Neurosci Lett 416(1):49–54 17300872 10.1016/j.neulet.2007.01.060

[37] Danzer SC (2019) Adult neurogenesis in the development of epilepsy. Epilepsy Curr 19:316–320 31409149 10.1177/1535759719868186 PMC6864561

